# Effect of Secular Trend, Age, and Length of Follow-up on Optimum Body Mass Index From 1985 Through 2015 in a Large Austrian Cohort

**DOI:** 10.2188/jea.JE20200012

**Published:** 2021-12-05

**Authors:** Raphael Simon Peter, Bernhard Föger, Hans Concin, Gabriele Nagel

**Affiliations:** 1 Institute of Epidemiology and Medical Biometry, Ulm University, Ulm, Germany; 2 Agency for Preventive and Social Medicine (aks), Bregenz, Austria

**Keywords:** BMI, mortality, age, secular trend, length of follow-up

## Abstract

**Background:**

Obesity and its health consequences will dominate health care systems in many countries during the next decades. However, the body mass index (BMI) optimum in relation to all-cause mortality is still a matter of debate.

**Material and Methods:**

Data of the Vorarlberg Health Monitoring & Prevention Program (VHM&PP, 1985–2005) and data provided by the Main Association of Austrian Social Security Institutions (MAASSI, 2005–2015) were analyzed. Information was available on age, sex, smoking status, measured height and weight, and mortality. Generalized additive models were used to model mortality as a function of BMI, calendar time, age, and follow-up.

**Results:**

In MAASSI (*N* = 282,216, 46.0% men), men and women were on average 2.7 years older than in VHM&PP (*N* = 185,361, 46.1% men). Average BMI was slightly higher in men (26.1 vs 25.7 kg/m^2^) but not in women (24.6 vs 24.7 kg/m^2^). We found an interactive effect of age and follow-up on the BMI optimum. Over age 35 years in men and 55 years in women, the BMI optimum decreased with length of follow-up. While keeping covariates fixed, BMI optimum increased slightly between 1985 and 2015 in men and women, 24.9 (95% CI, 23.9–25.9) to 26.4 (95% CI, 25.3–27.3), and 22.4 (95% CI, 21.7–23.1) to 23.3 (95% CI, 22.6–24.5) kg/m^2^, respectively.

**Conclusion:**

Age and length of follow-up have a pronounced effect on the BMI associated with the lowest all-cause mortality. After controlling for age and length of follow-up, the BMI optimum increased slightly over 30 years in this large study sample.

## INTRODUCTION

Obesity has become an explicit public health concern in high income and some middle-income countries.^1^ Recent studies indicate that obesity and its health consequences will dominate health care systems in many countries during the next decades.^1^^,^^2^ In children and adolescents, the increasing body mass index (BMI) trends have plateaued in many high-income countries, albeit at high levels.^2^ In Austria, the obesity prevalence is still rising, especially in men.^3^ Also, globally the increase in BMI has not slowed down.^1^

The association between BMI and mortality is, therefore, a matter of public interest.^4^^–^^7^ The relationship between BMI and mortality is U-shaped indication that low and high BMI is associated with higher mortality.^7^^,^^8^ The BMI optimum in the BMI all-cause mortality relationship changes with age and its pattern differs by sex.^6^ Heterogeneity in the association between BMI and mortality is attributable to ethnicity, age, and length of follow-up.^9^ Thus, these factors need consideration in the investigation of the BMI all-cause mortality relationship.

While average BMI increased over time, it is unclear if the BMI optimum stayed constant or increased as well.^4^ Afzal et al reported in 2016 that among three Danish cohorts, the BMI associated with the lowest mortality increased by 3.3 kg/m^2^ from 1976–1978 to 2003–2013.^4^ On the contrary, Wang et al found among Canadian adults that with fixed long-term follow-up duration, the BMI value associated with the lowest mortality remains relatively stable over time.^10^

While previous studies considered modifying factors like sex, age, length of follow-up,^9^ or a secular trend,^4^^,^^10^ there has not been an investigation into all these factors simultaneously. In this study, we, therefore, investigated the association of BMI with all-cause mortality in the general population over three decades considering a secular trend, sex, age, and length of follow-up as possible effect modifiers.

## METHODS

The Vorarlberg Health Monitoring & Prevention Program (VHM&PP) was carried out by the Agency of Social and Preventive Medicine in Vorarlberg, the westernmost Austrian state. The VHM&PP cohort has been described in detail previously.^5^ All adults in the state were invited to participate in a voluntary screening program of cardiovascular and malignant diseases. The costs were covered by the participants’ health insurance. The screening examination took place in the clinics of local physicians and included a physical examination, a blood test, and an interview by a physician. Between the 1^st^ of January 1985 and 30^th^ of June 2005, approximately 185,000 Vorarlberg residents aged 18 years and older were enrolled in the VHM&PP study cohort. The yearly participation rate was about 10%.

From August 1^st^, 2005 to December 31^st^, 2015, the Main Association of Austrian Social Security Institutions (MAASSI) in Vienna performed a screening program for cardiovascular diseases. Men and women aged 18 years and older were invited to participate in the program. The health examinations were conducted in 2- or 3-year intervals depending on age. The costs were covered by health insurance.

The basic program included an extensive discussion of the participant’s medical history, a physical examination by a doctor, and blood tests. In 2016, about 15% of the target population in Vorarlberg participated in the screening program.^11^

The VHM&PP data were merged with the MAASSI data of Vorarlberg participants in order to span the total period from 1985 through 2015. BMI was calculated from measured weight and height for both cohorts. Height and weight were measured in a standardized manner in both cohorts; participants did not wear shoes and had only light clothing. Height was measured by trained staff according to a standardized procedure with a precision of 1 cm and weight with a precision of 1 kg.

In Austria, every death is registered by the local registration office and transmitted to Statistic Austria, which administrates the national mortality registry. Both cohorts were linked to the national mortality registry to obtain the date of death for each deceased person. Due to different link time points, mortality information was available until December 2016 for VHM&PP and until August 2015 for MAASSI.

Ethical approval for the evaluation of the VHM&PP data was obtained from the ethics committee of Vorarlberg. For the MAASSI cohort, the ethical framework is covered by national law §459e ASVG.^12^ In the present study, the data were analyzed anonymously. The analyses of de-identified health care data are conformant with the Austrian law for data protection.^13^

### Data analysis

In cases where data of multiple examinations per person were available within one of the datasets, one examination of a person was chosen randomly and considered as the baseline in all subsequent analyses. The datasets were subsequently merged into one dataset, including the whole examination period from 1985 through 2015.

The merged dataset has been arranged in an aggregated manner using unique combinations (rows) of the variables baseline BMI, age, follow-up time, calendar time (all rounded to the nearest decile), sex, and baseline smoking status (ever vs never). Person-time at risk and the number of deaths have been calculated for each unique combination. Age, follow-up, and calendar time have been considered as time-varying variables with a resolution of 0.1 years. For example, a person followed for a total of 10 years contributed time under risk to 100 unique combinations with changing age, follow-up and calendar time, each increasing by an increment of 0.1 years at a time.

Generalized additive models (GAM) with a log link, Poisson distributed error term, and the log of person-time under risk as offset was used to model mortality as a function of calendar time, age, follow-up, and smoking. The GAM was set up using main effects (BMI, age, follow-up time, calendar time), two-way interaction terms (BMI * age, BMI * follow-up, BMI * calendar time, age * follow-up) and one three-way interaction term (BMI * age * follow-up). All terms were included as tensor product smooths.^14^ Also, all models included smoking as a covariate. Men and women were modeled separately. In addition, the above models were fit to never smokers only as a sensitivity analysis.

The BMI optimum for a specific combination of covariate values was determined numerically from the model as the BMI value for which the linear predictor (the log of the predicted mortality rate) reaches its minimum while holding the other covariate values constant. Corresponding confidence intervals were derived via posterior simulation based on the model parameter vector and the model covariance matrix.^15^ We performed 999 replications for confidence bands in figures and 999,999 replications for confidence intervals in tables. All analyses have been performed using R (R Foundation for Statistical Computing, Vienna, Austria). GAMs were fitted using the ‘bam’ function of the package ‘mgcv’ version 1.8–24, which is specifically suited to fit GAMs on very large data sets.

## RESULTS

In the present analyses, data of 467,577 participants were included. The VHM&PP cohort consists of 185,361 men and women and the MAASSI cohort of 282,216 Vorarlberg men and women, respectively (Table 1). Men and women in the MAASSI cohort were slightly older (mean age 48.0 vs 45.3 years in VHM&PP and 48.3 vs 45.6 years in the MAASSI cohort, respectively). In men, the average BMI was somewhat higher in the MAASSI cohort (26.1 vs 25.7 kg/m^2^), while in women, it was about the same (24.6 vs 24.7 kg/m^2^) as in the VHM&PP cohort. Also, the prevalence of ever smoker in the MAASSI cohort was lower for both men (22.4% vs 40.3%) and women (18.4% vs 24.8%) than in the VHM&PP cohort.

**Table 1.** Characterization of the study populations

	VHM&PP Jan. 1985–June 2005		MAASSI Aug. 2005–Dec. 2015	
	Male (*N* = 85,488)	Female (*N* = 99,873)	Male (*N* = 129,817)	Female (*N* = 152,399)
	
Age, years, mean (SD)	45.3 (15.5)	45.6 (16.6)	48.0 (16.9)	48.3 (17.7)

BMI, kg/m^2^, mean (SD)	25.7 (3.8)	24.7 (4.9)	26.1 (4.0)	24.6 (4.8)
BMI by WHO category, *N* (%)				
Underweight ≤18.5	827 (1.0)	4,245 (4.3)	1,212 (1.0)	6,386 (4.2)
Normal weight 18.5–<25	38,868 (45.5)	55,653 (55.7)	55,909 (43.1)	88,622 (58.2)
Overweight 25–<30	35,442 (41.5)	26,227 (26.3)	54,094 (41.6)	37,435 (24.6)
Obesity ≥30, *N*	10,351 (12.1)	13,748 (13.8)	18,602 (14.3)	19,956 (13.1)

Ever smoker, *N* (%)	34,441 (40.3)	24,721 (24.8)	29,020 (22.4)	28,068 (18.4)

Person-years of observation	1,544,094	1,889,757	365,145	435,102
Deaths	17,359	17,407	2,735	2,393
Deaths/1,000 person-years	11.2	9.2	7.5	5.5
Length of follow-up, years, median (Q1, Q3)	19.4 (14.8, 25.3)	20.1 (15.2, 25.8)	2.7 (1.3, 4.3)	2.7 (1.3, 4.3)

In the VHM&PP cohort, 17,359 deaths occurred among men during a median follow-up of 19.4 years and 17,407 deaths among women during a median follow-up of 20.1 years. In the MAASSI cohort, there were 2,735 deaths among men and 2,393 deaths among women during a median follow-up of 2.7 years.

**Figure 1.**
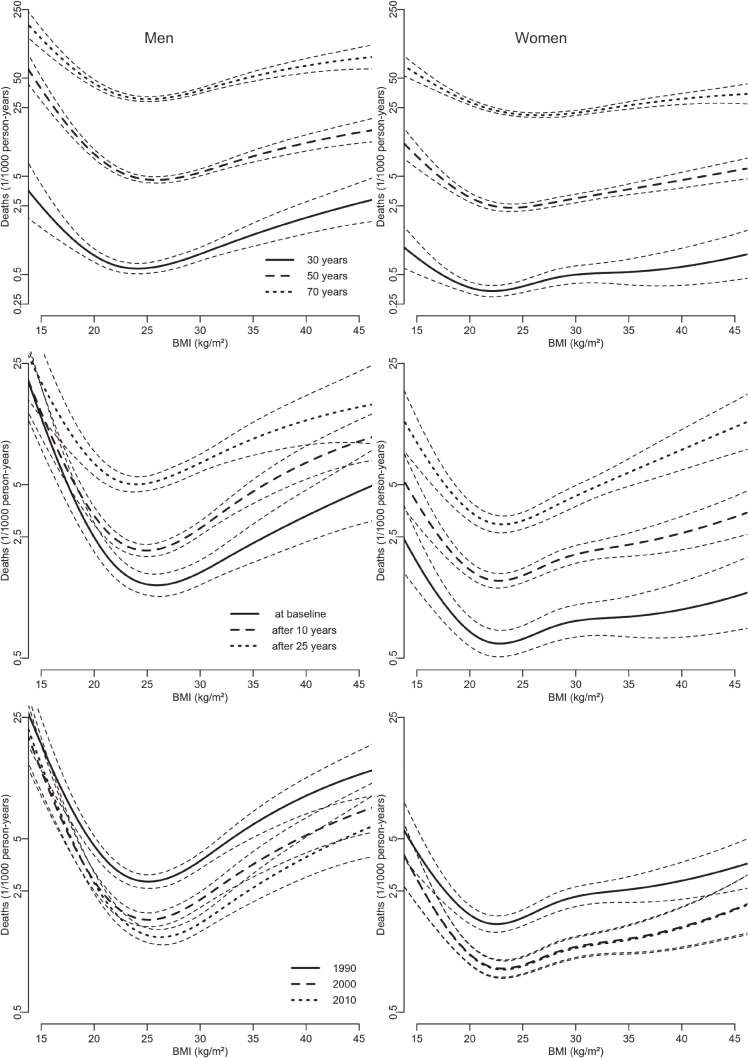
Mortality (deaths per 1,000 person-years) and 95% confidence bands by body mass index, according to age at baseline, follow-up year, and baseline year by sex (left men, right women) with covariates fixed at average values.

Figure 1 shows the shape of the BMI mortality association for the combined cohorts for specific years of age, follow up and calendar years times in men and women (each while keeping the others fixed at their average values). The association is clearly u-shaped in men and women of different age and at different follow-up times and calendar years. Optimum BMI (the position of the nadir of the curves) differs slightly by age and length of follow-up.

**Figure 2.**
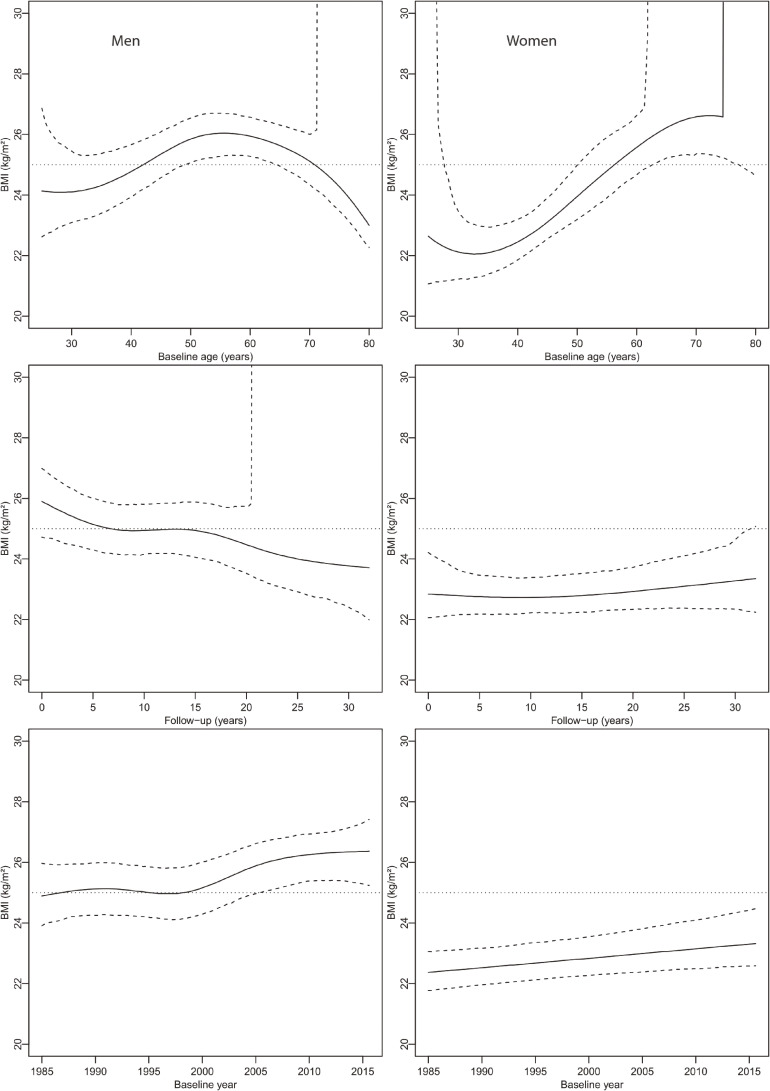
Body mass index optimum (in terms of minimum all-cause mortality) and 95% confidence bands according to age at baseline, follow-up year, and baseline year by sex (left men, right women) with covariates fixed at average values.

Figure 2 shows the optimum BMI according to age at baseline, follow-up time, and baseline year by sex. BMI optimum in men increased until the age of 58 years and decreased afterward. In women, BMI optimum increased with age as well. With the length of follow-up, the BMI optimum decreased in men from 25.9 to 23.7 kg/m^2^ after 30 years of follow-up while in women, the BMI optimum remained in the range of 23 kg/m^2^. In men, the BMI optimum increased from 24.9 (95% CI, 23.9–25.9) in 1985 to 26.4 (95% CI, 25.3–27.3) kg/m^2^ in 2015 and from 22.4 (95% CI, 21.7–23.1) to 23.3 (95% CI, 22.6–24.5) kg/m^2^ in women, respectively.

Sensitivity analysis in never smokers revealed similar patterns, albeit the optimum BMI was slightly lower at the same age and length of follow-up (eFigure 1).

We found evidence for an interaction between BMI, age, and follow-up (three-way interaction *P* = 0.001 in men and *P* < 0.001 in women). Table 2 shows BMI optima for selected combinations of age and length of follow-up by sex. For example, a man whose BMI is measured at 65 years of age would be at the lowest mortality risk after 10 years of follow-up (then at age 75 years) if he had a baseline BMI of 25.3 (95% CI, 24.6–26.1) kg/m^2^. Age and length of follow-up had a major impact on the BMI optimum. Up to the age of 30 years in men and 50 years in women, the BMI optimum was quite stable, independent of the length of follow-up. However, over 35 years in men and over 55 years in women, the BMI optimum decreased with length of follow-up, an interaction that became stronger with increasing age. At the age of 60 years, BMI optimum after 30 years of follow up was 22.5 kg/m^2^ in men and 23.1 kg/m^2^ in women. At the age of 70 years, for example, the BMI optimum decreased from 27.2 at baseline to 23.9 kg/m^2^ after 15 years of follow-up in men and from 27.4 to 24.3 kg/m^2^ in women, respectively.

**Table 2.** BMI optima and 95% confidence limits considering the interaction between age and length of follow-up by sex

Men							
Age at baseline, years	0	5	10	15	20	25	30
	years after baseline						
	
25	24.7 (22.6–37.6)	24.2 (22.6–29.2)	24.3 (22.7–26.9)	24.7 (22.8–31.0)	24.5 (22.6–NA)	24.3 (22.3–NA)	24.5 (22.0–NA)
30	24.7 (23.2–26.7)	24.2 (23.1–25.6)	24.2 (23.1–25.5)	24.4 (23.2–25.8)	24.1 (22.9–26.0)	23.8 (22.5–NA)	23.8 (22.1– NA)
35	25.0 (23.6–26.5)	24.4 (23.4–25.6)	24.3 (23.4–25.4)	24.4 (23.4–25.6)	24.1 (23.0–25.5)	23.7 (22.6–NA)	23.6 (22.1–NA)
40	25.6 (24.4–26.8)	24.9 (24.0–25.8)	24.8 (23.9–25.6)	24.8 (23.9–25.7)	24.3 (23.4–25.6)	23.9 (22.9–NA)	23.7 (22.3–NA)
45	26.4 (25.3–27.4)	25.5 (24.7–26.3)	25.3 (24.5–26.0)	25.3 (24.4–26.1)	24.7 (23.8–NA)	24.2 (23.2–NA)	23.9 (22.5–NA)
50	27.1 (26.1–28.1)	26.1 (25.3–26.8)	25.7 (25.0–26.4)	25.6 (24.8–26.5)	25.0 (24.0–NA)	24.3 (23.3–NA)	23.9 (22.4–NA)
55	27.4 (26.5–28.4)	26.3 (25.6–26.9)	25.9 (25.1–26.5)	25.7 (24.8–26.7)	24.9 (23.9–NA)	24.0 (23.0–NA)	23.4 (22.0–NA)
60	27.4 (26.6–28.3)	26.3 (25.6–26.8)	25.7 (25.1–26.3)	25.4 (24.6–NA)	24.4 (23.5–NA)	23.3 (22.3–NA)	22.5 (20.7–NA)
65	27.3 (26.5–28.2)	26.0 (25.3–26.6)	25.3 (24.6–26.1)	24.8 (24.0–NA)	23.5 (22.6–NA)	22.2 (20.6–NA)	NA (NA–NA)
70	27.2 (26.2–28.4)	25.6 (24.8–26.4)	24.7 (23.9–NA)	23.9 (22.9–NA)	22.2 (20.7–NA)	20.5 (NA–NA)	NA (NA–NA)
75	27.0 (25.7–NA)	24.9 (24.0–NA)	23.6 (22.8–NA)	22.3 (21.1–NA)	20.2 (NA–NA)	17.1 (NA–NA)	NA (NA–NA)
80	26.7 (24.9–NA)	23.8 (22.9–NA)	22.1 (21.1–NA)	20.1 (NA–NA)	15.8 (NA–NA)	NA (NA–NA)	NA (NA–NA)


Women							
Age at baseline, years	0	5	10	15	20	25	30
	years after baseline						
	
25	22.6 (15.2–NA)	22.6 (20.7–38.6)	22.6 (21.3–26.6)	22.7 (21.6–25.2)	22.9 (21.8–25.3)	23.3 (22.1–26.5)	23.8 (22.3–47.0)
30	22.1 (20.4–43.9)	22.1 (21.1–24.4)	22.1 (21.3–23.1)	22.1 (21.5–23.0)	22.4 (21.7–23.3)	22.7 (22.0–24.0)	23.2 (22.2–25.4)
35	22.1 (21.1–41.8)	22.1 (21.4–23.2)	22.1 (21.5–22.8)	22.1 (21.5–22.8)	22.3 (21.7–23.0)	22.6 (21.9–23.5)	23.0 (22.1–24.4)
40	22.6 (21.7–25.1)	22.5 (21.9–23.3)	22.4 (21.9–23.1)	22.5 (21.9–23.2)	22.6 (22.0–23.4)	22.8 (22.1–23.8)	23.1 (22.2–24.5)
45	23.2 (22.4–24.6)	23.1 (22.5–24.0)	23.1 (22.6–23.9)	23.2 (22.6–24.1)	23.4 (22.7–24.3)	23.5 (22.7–24.6)	23.5 (22.4–25.1)
50	24.0 (23.0–25.8)	23.9 (23.2–25.1)	24.0 (23.3–25.1)	24.2 (23.4–25.4)	24.3 (23.4–25.4)	24.2 (23.2–25.5)	24.0 (22.5–25.9)
55	24.9 (23.6–26.9)	24.8 (23.9–26.0)	24.8 (23.9–26.0)	25.0 (24.0–26.1)	24.9 (23.9–26.0)	24.6 (23.4–26.1)	24.0 (22.2–27.1)
60	25.9 (24.5–NA)	25.7 (24.7–26.9)	25.5 (24.6–26.5)	25.3 (24.3–26.4)	25.0 (23.9–26.5)	24.4 (22.8–NA)	23.1 (15.6–NA)
65	26.8 (25.3–NA)	26.4 (25.4–NA)	25.9 (24.9–NA)	25.2 (24.0–NA)	24.3 (22.8–NA)	22.4 (NA–NA)	18.3 (NA–NA)
70	27.4 (26.0–NA)	26.9 (25.9–NA)	26.0 (24.8–NA)	24.3 (22.8–NA)	21.4 (NA–NA)	NA (NA–NA)	NA (NA–NA)
75	27.6 (26.4–NA)	27.0 (26.1–NA)	NA (24.1–NA)	NA (NA–NA)	NA (NA–NA)	NA (NA–NA)	NA (NA–NA)
80	NA (26.6–NA)	NA (25.9–NA)	NA (22.3–NA)	NA (NA–NA)	NA (NA–NA)	NA (NA–NA)	NA (NA–NA)

## DISCUSSION

In this large population-based cohort study, including more than 450,000 participants, we found that from 1985 through 2015 the BMI optimum increased by approximately 1 kg/m^2^, after controlling for a changing age distribution and differences in length of follow-up. Age and length of follow-up itself had more pronounced effects on the BMI optimum; however, the effect of follow-up time was modified by age. Up to the age of 30 years in men and 50 years in women, the BMI optimum was similar over follow-up time, while in older age, the BMI optimum decreased with length of follow-up.

Our observation of an effect of age and length of follow-up on the BMI optimum is consistent with the literature.^6^^,^^16^ The interactive effect of age and length of follow-up might account for about 56% of the heterogeneity in BMI optima found in different studies.^9^

He (2009) found that BMI optimum in men depends on the follow-up time, while that for women depends on the age at BMI measurement.^16^ We found a relevant effect of both age and follow-up time in men and women. However, as in the analysis of He, changes in the BMI optimum with age were more pronounced in women. Also, the follow-up effect on the BMI optimum was stronger in men; and already relevant at a younger age. Mechanisms for differences in the BMI mortality associations between men and women are complex. The mechanisms include differences in lifestyle (eg, smoking prevalence, diet, and health care utilization) and biological factors like body fat distribution. Body fat distribution is an important determinant of the health consequences of obesity.^17^^,^^18^ Fat distribution differs between men and women and is changing with age.^19^ Also, genetic effects that influence fat distribution have been found to be stronger in women compared to men.^20^

Several (not necessarily mutually exclusive) explanations for an interactive effect of age and length of follow-up on the BMI optimum are plausible.

1. Individuals, on average, gain weight with aging,^21^ so baseline BMI usually underestimates BMI at later time points. Hence the estimated optimum would decrease with longer follow-up.
2. The stronger association between follow-up and BMI optimum in older participants could be explained by reverse causation (previous weight loss due to known or unknown disease),^22^ as the prevalence of chronic conditions increases with age; thus, the impact of reverse causation should be more pronounced in older subjects.
3. In older individuals, a higher BMI might represent a reserve in cachectic disease.^23^ However, this might provide mainly short-term benefit.

An increasing BMI optimum over the last decades has recently been of debate in the literature.^4^^,^^10^^,^^24^ Afzal et al reported a pronounced increase in BMI optimum of 3.3 kg/m^2^ in Danish cohorts from the years 1976 to 2013.^4^ Wang et al, on the other hand, found that when fixing the follow-up time, the BMI optimum remained relatively stable over time among American adults between 1986 and 2009.^10^ We found BMI optimum has increased slightly on average by 1 unit in men and 0.5 units in women from 1985 through 2015 in our cohorts.

The population age distribution changed in recent decades. Individuals included more recently in these studies have, on average shorter follow-up. As age and follow-up together are important determinants of the BMI optimum, it is crucial to adequately control these variables when investigating secular trends of the BMI optimum. We, therefore, modeled the effect of age, follow-up, age–follow-up interaction, and calendar time on the BMI optimum simultaneously within one single model.

The prevalence of obesity-related cardiovascular risk factors (eg, hypertension, hypercholesterolemia) decreased over recent decades,^25^^,^^26^ while detection and treatment may have improved. These may have reduced the hazard associated with higher BMI values and shifted the BMI optimum upwards, thereby leading to the observed secular trend.

Some limitations need to be kept in mind. Unfortunately, we could not take into account information on prevalent chronic diseases. BMI is an imperfect measure for adiposity, since BMI does not reflect the body compartments and the distribution fat of the body.^27^ However, the comparison between anthropometric measures and MRI revealed that in the age group 47 to 81 years, total and subcutaneous adipose tissue is highly correlated with BMI.^28^ Over time, the BMI optimum may have increased, but the higher BMI could be due to higher muscle mass. Also, higher BMI could have been associated with a favorable body fat distribution.^29^ When generalizing the results, it should be kept in mind that due to the self-referral of the prevention programs, the study sample is likely to be more health-conscious than the general population. The yearly participation rates where about 10% in VHM&PP and 15% for MASSI, however a substantial proportion of the Vorarlberg population participated at least once. For VHM&PP overall participation in the eligible age range has been reported as 55%.^5^

Our study has several strengths. We analyzed two large population-based cohorts in consecutive time intervals recruited in the same region. In both cohorts, height and weight were measured in a standardized manner. The observation time of this study covers 30 years with virtually complete follow-up. We did not attempt to control for factors like hypertension, diabetes, or blood lipids as we believe these factors might be on the causal pathway from obesity to mortality.

In conclusion, we found that age and length of follow up have a pronounced effect on the BMI associated with the lowest all-cause mortality. After controlling for age and length of follow-up, the BMI associated with the lowest all-cause mortality increased slightly over 30 years in this large study sample.

## APPENDIX A. SUPPLEMENTARY DATA

eFigure 1. Body mass index optimum (in terms of minimum all-cause mortality) and 95% confidence bands in never smokers according to age at baseline, follow-up year, and baseline year by sex (left men, right women) with covariates fixed at average values.
