# Supplementary material for: Effect of Secular Trend, Age, and Length of Follow-up on Optimum Body Mass Index From 1985 Through 2015 in a Large Austrian Cohort
Source: J Epidemiol. 2021 Dec 5;31(12):601–7. doi: 10.2188/jea.JE20200012 (PMC8593575; doi:10.2188/jea.JE20200012)
Supplement: Supplementary file 1 [file je-31-601-s001.pdf]

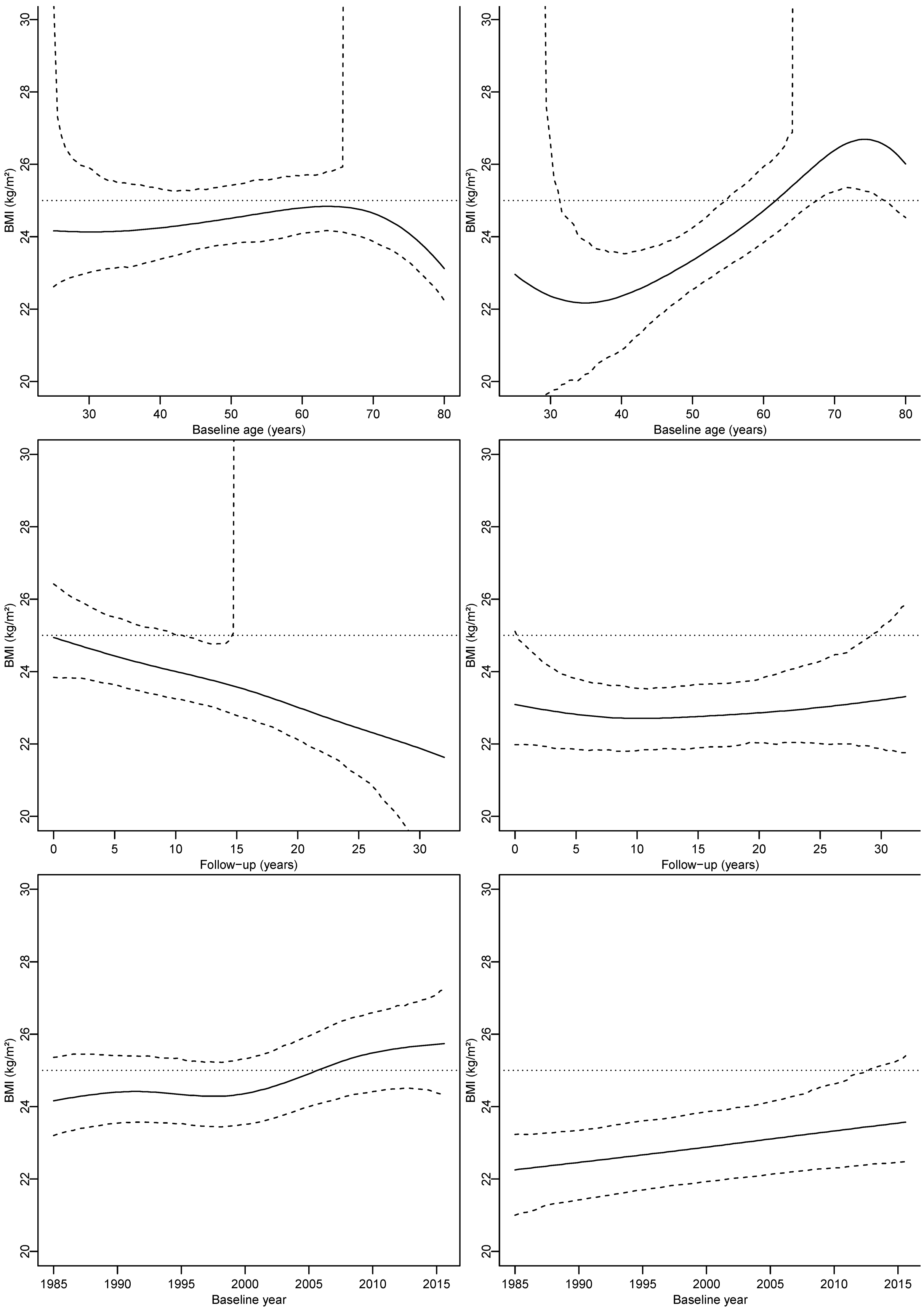


**eFigure 1.** Body mass index optimum (in terms of minimum all-cause mortality) and 95% confidence bands in never smokers according to age at baseline, follow-up year, and baseline year by sex (left men, right women) with covariates fixed at average values.
